# Octacalcium phosphate with incorporated carboxylate ions: a review

**DOI:** 10.1080/14686996.2022.2094728

**Published:** 2022-07-20

**Authors:** Taishi Yokoi, Masaya Shimabukuro, Masakazu Kawashita

**Affiliations:** Institute of Biomaterials and Bioengineering, Tokyo Medical and Dental University (TMDU), Tokyo, Japan

**Keywords:** Octacalcium phosphate, incorporation, carboxylate ions, biomaterials, functional materials

## Abstract

Octacalcium phosphate (OCP) belongs to a family of calcium phosphate compounds. OCP has unique crystal-chemical properties; among calcium phosphate compounds, only OCP can incorporate carboxylate ions into its crystal lattice. An OCP with incorporated carboxylate ions is called an OCP carboxylate (OCPC). OCPCs are investigated for applications in novel adsorbents, electrochemical devices, and biomaterials. Several wet methods are available for the synthesis of OCPCs, and the characteristics and advantages of each method are explained. Representative characterization methods, i.e. X-ray diffraction and Fourier transform infrared spectroscopy, used for the detection of carboxylate ion incorporation into the OCP interlayers are explained. Various carboxylic acids can be incorporated into OCP, and these types of carboxylic acid are presented with reference to the latest research results. The incorporation of carboxylate ions into OCP represents a modification of the OCP crystal at the molecular level and can impart various functions. Challenging physicochemical and biomaterial applications of OCPCs are thus introduced, although they are still in the research phase. Finally, future perspectives and challenges for OCPC research are described.

## GRAPHICAL ABSTRACT

**Figure:**
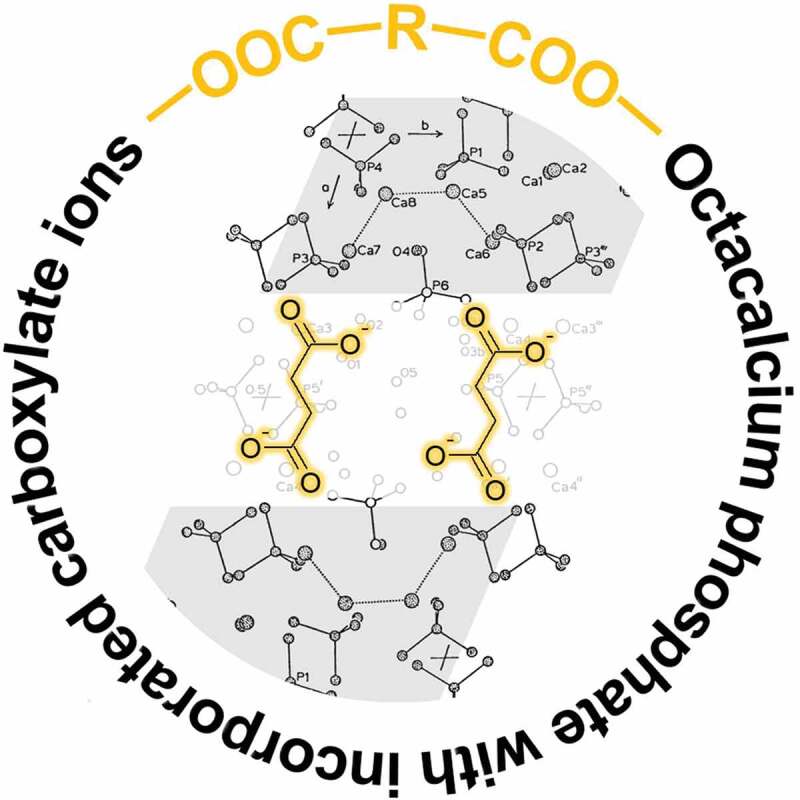

## 1. Introduction

Biomaterials science is an important and large research field due to the diversity of these materials. Biomaterials include polymers, metals, ceramics, and composites of these materials [1], and biomaterials science is a discipline that brings together the wisdom of materials science. For example, modern ceramic biomaterials originated with the development of bioactive glass, i.e. glass with bone-bonding properties, by Hench [2]. There was a time when bioactive glass research was active in Japan; however, such research, including glass-ceramic research, is now in decline. Bioactive inorganic/organic hybrid research [3,4] also ended following a temporary boom. On the other hand, research on calcium phosphate-based biomaterials, which began a little later than that on bioactive glass, remains one of the most important fields of ceramic biomaterials research [5], and calcium phosphate compounds have become indispensable in the study of biomaterials, especially ceramic-based biomaterials. Among calcium phosphate compounds, octacalcium phosphate (OCP) has unique properties in that it can incorporate carboxylate ions into its layered crystal structure. Only OCP exhibits such specific crystallographic properties, and we consider that these properties could lead to the development of various materials, including biomaterials. In this review, we will focus on calcium phosphate, particularly OCP with incorporated carboxylate ions, which is expected to be a next-generation material that will allow the flexible design of various functionalities.

## 2. Brief overview of calcium phosphate compounds

**Figure 1.**
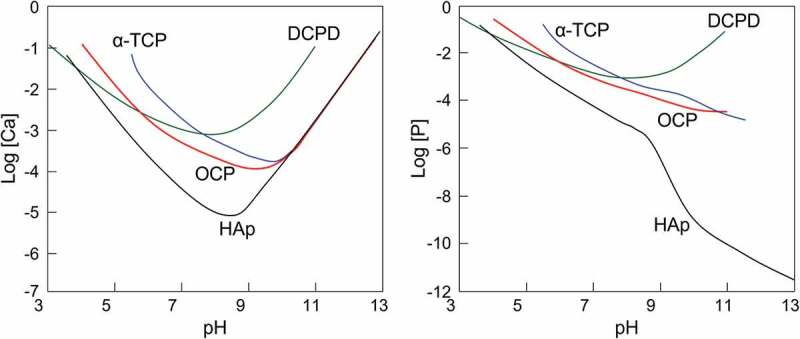
Solubility diagrams of representative calcium phosphate compounds at 37°C. Solubility isotherms showing (a) log[Ca] and (b) log[P] as a function of the solution pH. Reprinted from reference [6] with permission.

Phosphate ions can exist as mono- to trivalent anions and thus form compounds with calcium ions in various Ca/P molar ratios. Table 1 [5] shows a list of representative calcium phosphate compounds. A solubility diagram for calcium phosphate compounds with respect to pH is shown in Figure 1 [6]. The stable phases in aqueous solution are dicalcium phosphate dihydrate (DCPD) in an acidic environment, and hydroxyapatite (HAp) in neutral and alkaline environments. Therefore, metastable calcium phosphates in aqueous environments change to stable phases, and OCP is no exception. OCP is a metastable phase in aqueous solution, and it transforms into a stable phase depending on the pH of the aqueous solution. However, the transformation rate of OCP to a stable phase is generally not high, so that OCP can be obtained by wet chemical processes. In addition, OCP cannot be synthesized by dry methods because it contains crystalline water in its composition.

**Table 1.** Compositions and abbreviations for calcium phosphates [5].

Compound	Abbreviation	Formula	Ca/P molar ratio
Monocalcium phosphate monohydrate	MCPM	Ca(H_2_PO_4_)_2_·H_2_O	0.5
Monocalcium phosphate anhydrate	MCPA	Ca(H_2_PO_4_)_2_	0.5
Dicalcium phosphate dihydrate	DCPD	CaHPO_4_·2H_2_O	1.0
Dicalcium phosphate anhydrate	DCPA	CaHPO_4_	1.0
Octacalcium phosphate	OCP	Ca_8_(HPO_4_)_2_(PO_4_)_4_·5H_2_O	1.33
α-Tricalcium phosphate	α-TCP	Ca_3_(PO_4_)_2_	1.5
β-Tricalcium phosphate	β-TCP	Ca_3_(PO_4_)_2_	1.5
Amorphous calcium phosphate	ACP	Ca_*x*_(PO_4_)_*y*_·*n*H_2_O	1.2–2.2
Hydroxyapatite	HAp	Ca_10_(PO_4_)_6_(OH)_2_	1.67
Tetracalcium phosphate	TTCP	Ca_4_(PO_4_)_2_O	2.0

OCP can be a precursor phase for HAp in artificial synthetic systems [7,8]. OCP is also a precursor for biological apatite in bones [9] and is thus related to humans from a biological perspective and has the properties of an excellent bone repairing material [10,11]. The properties of OCP as a bone repairing material are well summarized in the book edited by Suzuki and Insley [12]; therefore, we refer the reader to that book.

A notable physicochemical property of OCP is that it can incorporate various carboxylate ions. Only OCP has this incorporation property among the calcium phosphates. From the next section, the unique incorporation behavior of OCP and the applications of these materials are introduced in terms of basic materials chemistry.

## 3. Octacalcium phosphate with incorporated carboxylate ions

**Figure 2.**
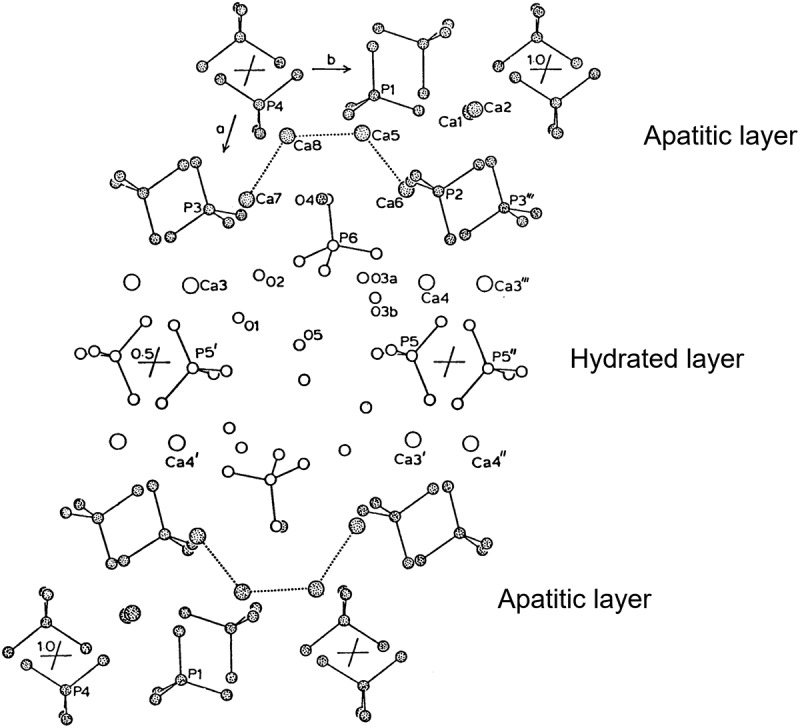
Crystal structure of OCP projected down from the *c*-axis direction. The shaded atoms correspond to the apatitic layer and the non-shaded atoms correspond to the hydrated layer. Reprinted from reference [13] with permission.

The space group and lattice constants for OCP are summarized in Table 2 and compared to HAp [5]. The atomic arrangement in OCP crystals is partially similar to that in HAp. However, OCP has characteristic properties that HAp does not have from a crystallographic perspective. A schematic illustration of the layered crystal structure of OCP is shown in Figure 2 [13]. The OCP crystal structure is composed of apatitic and hydrated layers, which are parallel to the (100) planes. The phosphate ions, PO_4_(1) and PO_4_(4), lie within the apatitic layer. The phosphate ions PO_4_(2), PO_4_(3), and the hydrogen phosphate ions HPO_4_(6) lie at the junctions of the apatitic and hydrated layers. The hydrogen phosphate ions HPO_4_(5) bridges the calcium ions within the hydrated layer. The HPO_4_(5) and HPO_4_(6) ions are not crystallographically equivalent. The detailed crystal structure and chemistry of OCP is explained in the literature [13–15]. It is important to note that HPO_4_(5) in the hydrated layers can be substituted by carboxylate ions. Among calcium phosphate compounds, only OCP can incorporate carboxylate ions into its crystal lattice; here, we focus on this unique incorporation property.

**Table 2.** Space groups and lattice constants for OCP and HAp [5].

Compound	Space group	Lattice constants	
		/nm	/degree
OCP	Triclinic *P$\overset{ˉ}{1}$*	*a* = 1.9692(4) *b* = 0.9523(2) *c* = 0.6835(2)	α = 90.15(2) β = 92.54(2) γ = 108.65(1)
HAp	Hexagonal *P*6_3_/m	*a*=*b* = 0.94302(5) *c* = 0.68911(2)	γ = 120

### 3.1. Synthesis

In 1983, Monma and Goto first reported the serendipitous discovery that HPO_4_^2^^‒^ groups in hydrated layers of OCP can be substituted by carboxylate ions [16]. A series of carboxylic acids that could be incorporated into OCP were discovered. It was subsequently determined that the carboxylic acids that could be incorporated into OCP were mainly dicarboxylic acids, which have two carboxy groups in one molecule [17,18]. The general formula for OCP with incorporated dicarboxylate ions is Ca_8_(HPO_4_)_2–*x*_(dicarboxylate ion)_*x*_(PO_4_)_4_·*y*H_2_O (0 < *x* ≤ 1). These compounds are called OCP carboxylates (OCPC). Hereafter, to clearly distinguish these compounds, OCP devoid of carboxylate ions is referred to as plain OCP.

While there are few reports of in-depth discussions on the kinetics for the formation of OCPCs [19], the process for the synthesis of OCP with incorporated carboxylate ions is typically simple. However, the process window may be narrower for some types of carboxylic acids. If the target carboxylic acid coexists under the synthesis conditions in which plain OCP is formed, then the OCPC is spontaneously formed. For example, OCPC is formed by the transformation of α-tricalcium phosphate (TCP) in target guest carboxylic acid solutions under weak acidic conditions. The synthesis of OCPC using α-TCP as a starting material is a typical method. This reaction is given by:

$$\begin{array}{rl} & 3Ca_{3}(PO_{4}{)}_{2}\;+\;xRC_{2}O_{4}^{2-}\;+\;(y+2)H_{2}O\;\\ & \;\;\;\;\;\;\;\;\;\;\;\to \;Ca_{8}(HPO_{4}{)}_{2-x}(RC_{2}O_{4}{)}_{x}(PO_{4}{)}_{4}\cdot yH_{2}O\\ & \;\;\;\;\;\;\;\;\;\;\;+\;x{HPO}_{4}^{2-}\;\;\;\;+\;\;\;Ca^{2+}\;\;\;+\;\;\;2OH^{-}\;(0\;<\;x\;\leq \;1) \end{array}$$

where RC_2_O_4_^2^^‒^ (R: hydrocarbon group, C_2_O_4_^2^^‒^ = 2(COO^‒^)) indicates a dicarboxylate ion. DCPD can be used as a starting material instead of α-TCP for the synthesis of OCPC [20]. The solubility of DCPD is less than that of α-TCP in the pH range where OCPC is formed; therefore, the process using DCPD is useful for the synthesis of OCP with incorporated carboxylate ions that react with calcium ions to form poorly soluble calcium salts. Other synthetic methods include the use of calcium carbonate as the calcium source and orthophosphoric acid solution as the phosphoric acid source [21]. In processes that use calcium phosphate compounds as starting materials, it is difficult to change the Ca/P molar ratio in the reaction system, whereas a synthesis process that uses calcium carbonate and phosphoric acid as starting materials has the advantage that the Ca/P molar ratio can be changed freely. However, the latter synthetic process may be unsuitable for some target carboxylic acid species due to large pH fluctuations in the reaction system during the synthesis. Therefore, the key to the success of OCPC synthesis is to select the appropriate synthetic process for the target carboxylic acid species.

### 3.2. Structure and composition

**Figure 3.**
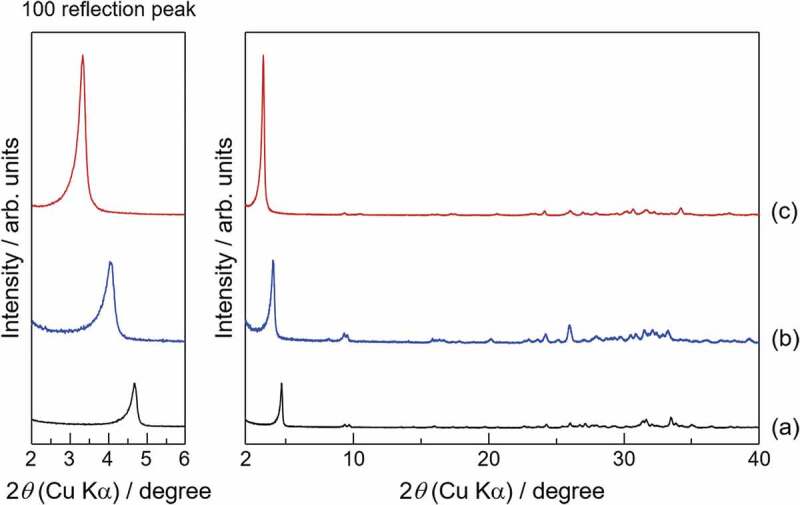
XRD patterns for (a) plain OCP, (b) OCP with incorporated succinate ions, and (c) OCP with incorporated suberate ions. Reprinted from reference [23] with permission.

**Figure 4.**
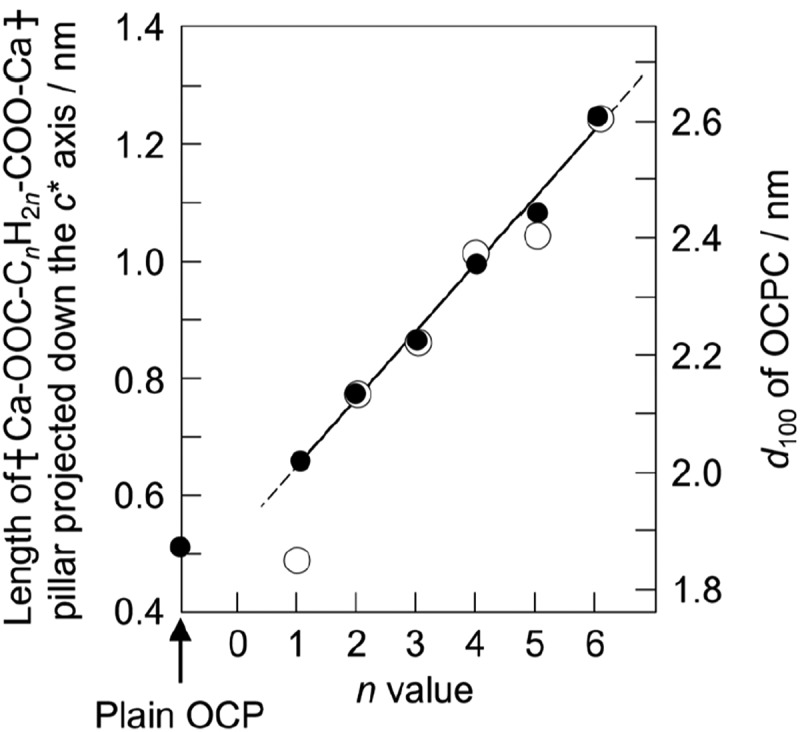
Relationship between Ca-OOC-C_*n*_H_2*n*_-COO-Ca length and *d*_100_ value for OCPCs. The filled and the open white circles indicate experimentally and graphically obtained values, respectively. c* axis: perpendicular to the *a-b* plane. Reprinted from reference [22] with permission.

It is necessary to confirm whether the target carboxylic acid has been successfully incorporated into OCP. Incorporation of carboxylate ions into OCP can be identified by X-ray diffraction (XRD), Fourier transform infrared (FTIR) spectroscopy, Raman scattering, solid-state nuclear magnetic resonance (NMR), and compositional analysis. Among these analytical methods, XRD is the most representative and effective because the incorporation of carboxylate ions into OCP involves significant changes of the (100) interplanar spacing (*d*_100_) of OCP. The interplanar spacings (*d*) for OCP incorporated with aliphatic dicarboxylate ions such as succinate, adipate, and suberate ions, are summarized in Table 3 and compared with those for plain OCP [22]. The *d*_100_ value for OCP increases with the molecular size of the incorporated dicarboxylate ion, whereas the changes in *d*_010_ and *d*_002_ are small because the incorporated dicarboxylate ions lie parallel to the *a*-axis. Therefore, as shown in Figure 3 [23], the incorporation of carboxylate ions into OCP shifts the 100 reflection peak for OCP significantly to lower angles. In addition, as shown in Figure 4 [22], Monma noticed a relationship between *d*_100_ for OCPC and the molecular size of the incorporated carboxylic acid since the initial stages of OCPC research. The molecular sizes were obtained geometrically and the relationships shown in Figure 4 were determined with a high degree of accuracy. We employed a computational chemistry approach and confirmed the validity of the prediction by Monma, in that the aliphatic carboxylic acids are present in the interlayers of OCP while maintaining a linear structure [24]. One new possibility was also observed in that some carboxylic acids with side chains may be incorporated into the interlayers of OCP with a slightly bent conformation. It is important to emphasize here that the interlayer distance in OCP, a layered compound, can be precisely controlled at the nanometer level by the size of the guest carboxylate ions incorporated into the OCP interlayers.

**Table 3.** Interplanar spacings for plain OCP and OCPCs [22].

Incorporated anions	Interplanar spacings/nm		
	*d*_100_	*d*_010_	*d*_002_
Hydrogen phosphate	1.87	0.936	0.342
Succinate	2.14	0.939	0.342
Adipate	2.36	0.941	0.342
Suberate	2.61	0.938	0.342

**Figure 5.**
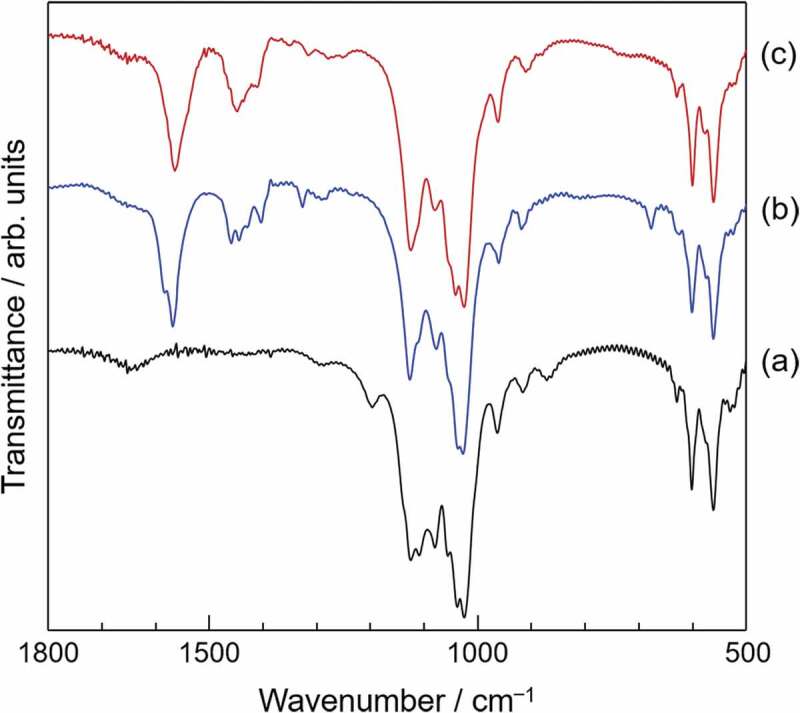
FTIR spectra of (a) plain OCP, (b) OCP with incorporated succinate ions and (c) OCP with incorporated suberate ions. Reprinted from reference [23] with permission.

FTIR spectroscopy is an appropriate analytical method to determine the incorporation of carboxylate ions into OCP. The absorption peak derived from hydrogen phosphate ions in the hydrated layer (HPO_4_(5) in Figure 2) is detected at 1193 cm^‒^^1^. This absorption peak disappears after incorporation of carboxylate ions into OCP due to the substitution of the hydrogen phosphate ions by dicarboxylate ions. Absorption peaks derived from incorporated carboxylate ions appear instead. Figure 5 [23] shows FTIR spectra of plain OCP and representative OCPCs. Raman spectroscopic analysis is also a useful method to determine the incorporation of carboxylate ions into OCP. The peak assignments in FTIR and Raman spectra have been described precisely in a previous report [25,26]. In association with these analytical methods, solid-state NMR spectroscopy is also useful for identifying the incorporation of carboxylate ions into OCP. NMR-based OCPC studies have been conducted [27–29]. ^31^P NMR analysis can indicate substitution with carboxylate ions by the disappearance of the peak derived from hydrogen phosphate ions (HPO_4_(5)). In addition, solid-state NMR can be used to study the chemical state of incorporated carboxylate ions by ^13^C analysis and provides important results on the structure of the carboxylate ions in the interlayers. In addition, ^43^Ca NMR analysis can be applied to investigate the structure of the hydrated layers. However, solid-state NMR is not a method used to analyze the crystal structure itself; therefore, it is necessary to characterize the crystal structure of the OCPC using XRD and neutron diffraction in the future.

Compositional analysis provides strong evidence of the incorporation of carboxylate ions into OCP by the substitution of hydrogen phosphate ions; the Ca/P molar ratio for OCPC is higher than that for plain OCP. In the case of dicarboxylate ions, the Ca/P molar ratio for OCPC ranges from 1.33 to 1.60. The general formula for OCPC containing dicarboxylate ion is Ca_8_(HPO_4_)_2‒*x*_(dicarboxylate ion)_*x*_(PO_4_)_4_·*y*H_2_O; therefore, if the Ca/P molar ratio for OCPC is known, then the substitution ratio *x* for hydrogen phosphate ions by dicarboxylic acids can be determined. Carbon content analysis also provides unequivocal evidence of the incorporation of carboxylate ions into OCP. This method can directly measure the carbon content derived from incorporated carboxylate ions in OCPC. However, because it is difficult to accurately measure the water content of OCP, i.e. *y*, in the general formula for OCPC, the results of carbon content analysis should be considered as semi-quantitative. The difficulties in measuring the water content of OCPC have previously been described [23].

### 3.3. Carboxylate ions that can be incorporated into OCP

The carboxylic acids used in the formation of OCPC are summarized in Table 4 [16–18,20–22,27,30–48]. The details have been previously reported [23]; therefore, we will give only a brief overview of the carboxylic acids that can be incorporated into OCP and then examine the latest research results since the initial report. The characteristics of carboxylic acids that can be incorporated into OCP are:

1. Monocarboxylic acids basically cannot be incorporated into OCP.
2. Saturated dicarboxylic acids without side chains can likely be incorporated into OCP.
3. For unsaturated dicarboxylic acids, some can be incorporated into OCP and some cannot. The factors that govern the incorporation behavior are not well understood.
4. Aromatic dicarboxylic acids can likely be incorporated into OCP.
5. For dicarboxylic acids with side chains, some can be incorporated into OCP and some cannot. The factors that govern the incorporation behavior are not well understood.
6. The only tricarboxylic acid that can be incorporated into OCP is citric acid.

**Table 4.** Carboxylic acids used in the formation of OCPC.

Carboxylic acid (CAS No.)	Formula	Molecular weight	OCPC formation	Year [Reference No.]
*Monocarboxylic acids*				
Formic acid (64-18-6)	HCOOH	46.03	No	1992 [48] 1993 [17] 2001 [45]
Acetic acid (64-19-7)	CH_3_COOH	60.05	No	1984 [18] 1992 [48] 1993 [17] 2001 [45]
Propionic acid (79-09-4)	CH_3_CH_2_COOH	74.08	No	2011 [41]
Glycine (56-40-6)	NH_2_CH_2_COOH	75.07	No	1992 [48] 1993 [17] 2000 [47] 2001 [45]
Pyruvic acid (127-17-3)	CH_3_COCOOH	88.06	Yes	1993 [17]* 2001 [45]*
Lactic acid**	CH_3_CH(OH)COOH	90.08	No	1992 [48]
DL-2-Aminobutyric acid (2835-81-6)	CH_3_CH_2_CH(NH_2_)COOH	103.12	No	2000 [47]
DL-Norvaline (760-78-1)	CH_3_(CH_2_)_2_CH(NH_2_)COOH	117.15	No	2000 [47]
D-Norleucine (327-56-0)	CH_3_(CH_2_)_3_CH(NH_2_)COOH	131.18	No	2000 [47]
L-Norleucine (327-57-1)	CH_3_(CH_2_)_3_CH(NH_2_)COOH	131.18	No	2000 [47]
DL-Norleucine (616-06-8)	CH_3_(CH_2_)_3_CH(NH_2_)COOH	131.18	No	2000 [47]
L-Ornithine (70-26-8)	NH_2_(CH_2_)_3_CH(NH_2_)COOH	132.16	No	2000 [47]
L-Glutamine (56-85-9)	NH_2_CO(CH_2_)_2_CH(NH_2_)COOH	146.15	No	2000 [47]
L-Lysine (56-87-1)	NH_2_(CH_2_)_4_CH(NH_2_)COOH	146.19	No	2000 [47]
*Saturated dicarboxylic acid*				
Malonic acid (141-82-2)	HOOCCH_2_COOH	104.06	Yes	1984 [18] 1984 [22] 1992 [48] 1993 [17] 2001 [45]
Succinic acid (110-15-6)	HOOC(CH_2_)_2_COOH	118.09	Yes	1983 [16] 1984 [18] 1984 [22] 1992 [48] 1993 [17] 2000 [46] 2001 [44] 2001 [45] 2008 [21] 2008 [43] 2010 [42] 2012 [39] 2012 [40] 2018 [20] 2021 [31]
Glutaric acid (110-94-1)	HOOC(CH_2_)_3_COOH	132.12	Yes	1984 [18] 1984 [22] 1992 [48] 2001 [45]
Adipic acid (124-04-9)	HOOC(CH_2_)_4_COOH	146.14	Yes	1984 [18] 1984 [22] 1992 [48] 1993 [17] 2001 [45] 2008 [21]
Pimelic acid (111-16-0)	HOOC(CH_2_)_5_COOH	160.17	Yes	1984 [18] 1984 [22] 1992 [48] 2001 [45]
Suberic acid (505-48-6)	HOOC(CH_2_)_6_COOH	174.20	Yes	1984 [18] 1984 [22] 1992 [48] 1993 [17] 2001 [45] 2008 [21] 2012 [39] 2012 [40]
			No	2018 [20]
Azelaic acid (123-99-9)	HOOC(CH_2_)_7_COOH	188.22	Yes	1992 [48]
			No	1993 [17] 2001 [45]
Sebacic acid (111-20-6)	HOOC(CH_2_)_8_COOH	202.25	Yes	1992 [48] 1993 [17] 2001 [45]
*Unsaturated dicarboxylic acid*				
Maleic acid (110-16-7)	HOOCCH=CHCOOH	116.07	Yes	1992 [48]
			No	1993 [17] 2001 [45]
Fumaric acid (110-17-8)	HOOCCH=CHCOOH	116.07	Yes	1984 [18] 1992 [48] 1993 [17] 2001 [45]
Citraconic acid (498-23-7)	HOOCC(CH_3_)=CHCOOH	130.10	Yes	1984 [18] 1992 [48]
Mesaconic acid (498-24-8)	HOOCC(CH_3_)=CHCOOH	130.10	No	1992 [48]
Itaconic acid (97-65-4)	HOOCCH_2_C(=CH_2_)COOH	130.10	No	1992 [48]
*cis, cis*-Muconic acid (1119-72-8)	HOOCCH=CHCH=CHCOOH	142.11	Yes	1992 [48]
*trans, trans*-Muconic acid (3588-17-8)	HOOCCH=CHCH=CHCOOH	142.11	No	1992 [48]
β-Dihydromuconic acid (4436-74-2)	HOOCCH_2_CH=CHCH_2_COOH	144.13	Yes	1984 [18] 1992 [48]
*Aromatic dicarboxylic acid*				
Phthalic acid (88-99-3)	HOOC(C_6_H_4_)COOH	166.13	Yes	1984 [18] 1992 [48] 2013 [38]
Isophthalic acid (121-91-5)	HOOC(C_6_H_4_)COOH	166.13	Yes	1992 [48] 2013 [38] 2018 [20] 2022 [30]
Terephthalic acid (100-21-0)	HOOC(C_6_H_4_)COOH	166.13	No	1992 [48] 2013 [38]
2,2’-Bipyridine-5,5’-dicarboxylic acid (1802-30-8)	HOOC(C_5_H_3_N)_2_COOH	244.21	Yes	2019 [34]
*Dicarboxylic acid with side chain*				
L-Malic acid (97-67-6)	HOOCCH_2_CH(OH)COOH	134.09	Yes	1993 [17] 2001 [45]
DL-Malic acid (6915-15-7)		134.09	Yes	2000 [46]
Malic acid**		134.09	No	1992 [48]
Mercaptosuccinic acid**	HOOCCH_2_CH(SH)COOH	150.15	Yes	2000 [46] 2015 [36] 2019 [33]
(*S*)-(–)-Methylsuccinic acid (2174-58-5)	HOOCCH_2_CH(CH)_3_COOH	132.12	Yes	2017 [35]
(*R*)-(+)-Methylsuccinic acid (3641-51-8)	HOOCCH_2_CH(CH)_3_COOH	132.12	No	2017 [35]
Methylsuccinic acid**	HOOCCH_2_CH(CH)_3_COOH	132.12	Yes	1984 [18] 1992 [48] 2000 [46] 2001 [44]
D-Aspartic acid (1783-96-6)	HOOCCH_2_CH(NH_2_)COOH	133.10	Yes	2000 [47]
L-Aspartic acid (56-84-8)	HOOCCH_2_CH(NH_2_)COOH	133.10	Yes	2000 [46] 2000 [47] 2001 [44]
DL-Aspartic acid (617-45-8)	HOOCCH_2_CH(NH_2_)COOH	133.10	Yes	2000 [47]
Aspartic acid**	HOOCCH_2_CH(NH_2_)COOH	133.10	Yes	2008 [43]
			No	1992 [48] 1993 [17] 2001 [45]
D-Glutamic acid (6893-26-1)	HOOCCH_2_CH_2_CH(NH_2_)COOH	147.13	Yes	2000 [47]
L-Glutamic acid (56-86-0)	HOOCCH_2_CH_2_CH(NH_2_)COOH	147.13	Yes	2000 [47]
DL-Glutamic acid (617-65-2)	HOOCCH_2_CH_2_CH(NH_2_)COOH	147.13	Yes	2000 [47]
Glutamic acid**	HOOCCH_2_CH_2_CH(NH_2_)COOH	147.13	No	1992 [48] 1993 [17] 2001 [45] 2008 [43]
*Other dicarboxylic acid*				
Oxalic acid (144-62-7)	HOOCCOOH	90.03	No	1984 [18] 1992 [48] 2001 [45]
Oxaloacetic acid (328-42-7)	HOOCCH_2_COCOOH	132.07	No	1993 [17] 2001 [45]
α-Ketoglutaric acid (328-50-7)	HOOC(CH_2_)_2_COCOOH	146.10	Yes	1993 [17]* 2001 [45]*
β-Ketoglutaric acid (542-05-2)	HOOCCH_2_COCH_2_COOH	146.10	No	1993 [17] 2001 [45]
*Tricarboxylic acid*				
Citric acid (77-92-9)	HOOCCH_2_C(OH)(COOH)CH_2_COOH	192.12	Yes	1993 [17] 2001 [45] 2008 [43] 2014 [37] 2021 [27]
			No	1992 [48]
*Tetracarboxylic acid*				
Pyromellitic acid (89-05-4)	(C_6_H_2_)(COOH)_4_	254.15	Yes	2021 [32]
*Others*				
Urea (57-13-6)	NH_2_CONH_2_	60.06	No	1992 [48]
Hydroquinone (123-31-9)	HO(C_6_H_4_)OH	110.11	No	1992 [48]
1,2-Ethanedisulfonic acid (110-04-3)	HO_3_S(CH_2_)_2_SO_3_H	190.18	No	1992 [48]
m-Benzenedisulfonic acid (98-48-6)	HO_3_S(C_6_H_4_)SO_3_H	238.24	No	1992 [48]

*The chemical species of the guest molecule in OCPC is uncertain.

**No description with respect to steric configuration of carboxylic acid.

Many of the carboxylic acids that are now known to be incorporated into OCP were found by Monma and Goto [18,48] and by Marković and colleagues [17,45]. Five major discoveries have been made in recent years. The first is that it is possible to simultaneously incorporate two types of carboxylate ions into OCP [40]. OCPs with various *d*_100_ values can be obtained by changing the ratio of the two types of carboxylate ions incorporated into OCP. The second is the discovery of a carboxylic acid (mercaptosuccinic acid) that continuously increases *d*_100_ for OCP [36]. The first and second findings are very useful for control of *d*_100_ for OCP at the nanometer level. The third finding is that enantioselective incorporation by OCP was reported in 2017 [35]; OCP incorporated (*S*)-(−)-methylsuccinic acid but no (*R*)-(+)-methylsuccinic acid was incorporated. This phenomenon clearly indicates that OCP recognizes the steric structures of guest molecules. Such a phenomenon is rare and not observed during general intercalation in layered compounds. The fourth finding is that the carboxylic acid that produced the largest interlayer distance of OCPC was suberic acid; however, 2,2’-bipyridine-5,5’-dicarboxylic acid was reported to produce OCPC with a larger interlayer distance in 2019 [34]. While *d*_100_ for OCP with incorporated suberate ions was 2.6 nm, that for OCP with incorporated 2,2’-bipyridine-5,5’-dicarboxylate ions was 2.7 nm. Although the difference is only 0.1 nm, this finding is significant in that it transcended the maximum value of *d*_100_ for OCPCs. The fifth finding is the discovery in 2021 that a tetracarboxylic acid (pyromellitic acid) could be incorporated into OCP [32]. Carboxylic acids that can be incorporated into OCP are basically dicarboxylic acids, and citric acid, a tricarboxylic acid, has been considered an exception. The successful incorporation of a tetracarboxylic acid into OCP likely means that there is still much room for exploration of carboxylic acids that can be incorporated into OCP.

There are 25 types of carboxylic acids that can be incorporated into OCP. (Note that the steric structure of the carboxylic acid is not considered here.) With regard to molecular weight, the value of 254 for pyromellitic acid is the largest, followed by 244 for 2,2’-bipyridine-5,5’-dicarboxylic acid. We expect that the number of guest molecular species that can be incorporated into OCP will continue to increase and that the incorporation of even larger molecular weight molecules will be reported.

### 3.4. Applications

OCPCs have not yet been used as practical materials, and both their physicochemical and biological applications are still in the research stage. It has been reported that precise control of the layered structure of OCP at the molecular level using carboxylate ions would enable the application OCPCs as novel functional materials.

From a physicochemical viewpoint, the first reported application of OCPCs was as a specific adsorbent for aldehydes [49]. The aldehydes reacted with aspartate ions incorporated into the OCP interlayers to form imines and were immobilized. Tuncer et al. recently reported the capacitive behavior of OCP synthesized in a solution containing succinic acid as an electrode material for supercapacitors [50]. Various designs of asymmetric and symmetric capacitors were prepared using the OCP powder as a potential electrode material. Each sample exhibited typical pseudocapacitive behavior. Electrochemical impedance spectra of the OCP materials confirmed their significant capacitive performance. Tuncer et al. suggested that this may be valuable for future medical electronics applications, such as biocompatible energy storage and energy harvesting microdevices.

The application of plain OCP as a bone-repairing material has been widely studied. Kawai et al. reported bone regeneration using an OCP-collagen composite for human bone defects as a first clinical trial [51]. The composite was implanted into bone defects after operations on patients with radicular cysts or apical periodontitis. Postoperative wound healing was uneventful, and neither infection nor allergic reaction against the composite was observed. Although the application of OCPCs as a bone-repairing material has potential, there are very few reported studies on the biological properties of OCPCs. In 2009, Ishihara et al. investigated the reaction of OCP with incorporated adipate ions in a type of simulated body fluid (SBF) and reported that OCPCs are promising candidates as bone-repairing materials [52]. An accelerated test study of the reactivity of OCPCs in aqueous solution has shown that the reactivity of OCPCs increases with increasing *d*_100_ [53]. Therefore, the incorporation of carboxylate ions is likely available as a reactivity controlling parameter of OCP *in vivo*.

**Figure 6.**
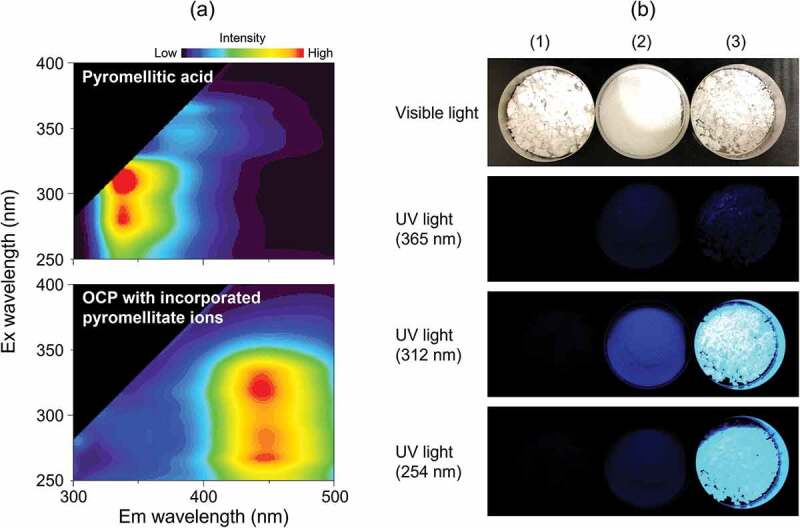
(a) 3D fluorescent spectra of pyromellitic acid and OCP with incorporated pyromellitate ions, and (b) images of samples under visible and UV light (365, 312, and 254 nm); (1) plain OCP, (2) pyromellitic acid and (c) OCP with incorporated pyromellitate ions. Reprinted from reference [32] with permission. .

The development of new biomaterials that utilize the function of carboxylic acids incorporated into OCP is also being studied. Fluorescent properties can be imparted to OCP by the incorporation of aromatic carboxylate ions, such as 2,2’-bipyridine-5,5’-dicarboxylate [34], isophthalate [30] and pyromellitate ions [32]. A 3D fluorescent spectrum of OCP with incorporated pyromellitate ions is shown in Figure 6. Although the fluorescent center is not a carboxylate ion, OCP with incorporated succinate ions doped with Eu^3+^ has also been investigated as a phosphor [54]. These are potential theranostic materials that enable therapy and diagnosis, and could be revolutionary materials that enable fluorescent diagnosis and bone regeneration. However, knowledge of the biological properties of OCPCs is definitely lacking. Therefore, basic research on the *in vitro* and *in vivo* behavior of OCPCs is necessary for their use in the development of novel biomaterials.

## 4. Future perspectives

A major issue related to research on OCPCs is that the detailed crystal structures are unknown. In addition to the difficulty of obtaining single crystals that are sufficiently large for crystal structure analysis, the complexity of the OCPC crystal structure is a serious barrier to it elucidation. This barrier will have to be overcome for the development of materials using OCPCs. It is also necessary to establish a reliable method for quantitative analysis of the composition, especially the amount of crystalline water. Overcoming these barriers is expected to lead to the establishment of a method for the incorporation of anions other than carboxylate ions into OCP, or to an understanding of the reasons for the difficulty of incorporating anions other than carboxylate ions into OCP.

In addition, plain OCP has a high affinity with hard tissues and as such, OCPCs would also likely have this affinity and are considered suitable for use as biomaterials. It is now being demonstrated that OCPCs can have functionalities that plain OCP does not have by the incorporation of carboxylate ions. Materials scientists are expected to attempt the development of functional OCPCs with an eye toward clinical applications.

## 5. Conclusions

In this review, we have summarized the methods of synthesis of OCP with incorporated various carboxylate ions, their structures and compositions, the applications of the resultant OCPCs, and future perspectives with OCPC research. We have introduced the developmental challenges for specific adsorbents, bone-repairing materials, and biofriendly capacitors and fluorescent materials by the incorporation of carboxylate ions into OCP to control the structure at the molecular level. Even though OCPCs have been known since 1983, they are probably the least studied of the calcium phosphate compounds. There are thus broad unexplored areas for OCPC research. OCPCs that express novel functions are expected to be developed in the near future by the modification of OCP crystals with functional carboxylic acids. We sincerely hope that this review will arouse the interest of many researchers and contribute in some way to the progress of research on OCPCs.

## References

[1] Park J, Lakes RS. Biomaterials: an introduction. 3rd ed. New York (NY): Springer; 2007.

[2] Hench LL. Bioceramics. J Am Ceram Soc. 1998;81:1705–1728.

[3] Ohtsuki C, Miyazaki T, Tanihara M. Development of bioactive organic–inorganic hybrid for bone substitutes. Mater Sci Eng C. 2002;22(1):27–34.

[4] Yabuta T, Tsuru K, Hayakawa S, et al. Synthesis of bioactive organic-inorganic hybrids with γ-methacryloxypropyltrimethoxysilane. J SoL-Gel Sci Technol. 2000;19:745–748.

[5] Dorozhkin SV, Epple M. Biological and medical significance of calcium phosphates. Angew Chem Int Ed. 2002;41:3130–3314. 10.1002/1521-3773(20020902)41:17<3130::AID-ANIE3130>3.0.CO;2-1 12207375

[6] Chow LC. Development of self-setting calcium phosphate cements. J Ceram Soc Jpn. 1991;99:954–964.

[7] Ito N, Kamitakahara M, Murakami S, et al. Hydrothermal synthesis and characterization of hydroxyapatite from octacalcium phosphate. J Ceram Soc Jpn. 2010;118:762–766.

[8] Kamitakahara M, Ito N, Murakami S, et al. Hydrothermal synthesis of hydroxyapatite from octacalcium phosphate: effect of hydrothermal temperature. J Ceram Soc Jpn. 2009;117:385–387.

[9] Crane NJ, Popescu V, Morris MD, et al. Raman spectroscopic evidence for octacalcium phosphate and other transient mineral species deposited during intramembranous mineralization. Bone. 2006;39(3):434–442. doi:10.1016/j.bone.2006.02.059 16627026

[10] Hamai R, Sakai S, Shiwaku Y, et al. Octacalcium phosphate crystals including a higher density dislocation improve its materials osteogenecity. Appl Mater Today. 2022;26:101279.

[11] Okuyama K, Shiwaku Y, Hamai R, et al. Differentiation of committed osteoblast progenitors by octacalcium phosphate compared to calcium-deficient hydroxyapatite in Lepr-cre/tomato mouse tibia. Acta Biomater. 2022;142:332–344. 35183778 10.1016/j.actbio.2022.02.016

[12] Suzuki O, Insley G. Octacalcium phosphate biomaterials: understanding of bioactive properties and application. Duxford (UK): Woodhead Publishing; 2020.

[13] Mathew M, Brown WE. A structural model for octacalcium phosphate–succinate double salt. Bull Chem Soc Jpn. 1987;60(3):1141–1143.

[14] Elliotto JC. Structure and chemistry of the apatites and other calcium orthophosphates. Amsterdam (Netherlands): Elsevier; 1994.

[15] Kanazawa T. Inorganic phosphate materials. Amsterdam (Netherlands): Elsevier; 1989.

[16] Monma H, Goto M. Succinate-Complexed octacalcium phosphate. Bull Chem Soc Jpn. 1983;56:3843–3844.

[17] Marković M, Fowler BO, Brown WE. Octacalcium phosphate carboxylates. 1. Preparation and identification. Chem Mater. 1993;5:1401–1405.

[18] Monma H, Goto M. Complexes of apatitic layered compound Ca_8_(HPO_4_)_2_(PO_4_)_4_⋅5H_2_O with dicarboxylates. J Inclusion Phenom. 1984;2:127–134.

[19] Marković M, Fowler BO, Brown WE. Octacalcium phosphate carboxylates. IV. Kinetics of formation and solubility of octacalcium phosphate succinate. J Cryst Growth. 1994;135(3–4):533–538.

[20] Yokoi T, Goto T, Kitaoka S. Transformation of dicalcium phosphate dihydrate into octacalcium phosphate with incorporated dicarboxylate ions. J Ceram Soc Jpn. 2018;126:462–468.

[21] Kamitakahara M, Okano H, and Tanihara M, et al. Synthesis of octacalcium phosphate intercalated with dicarboxylate ions from calcium carbonate and phosphoric acid. J Ceram Soc Jpn. 2008;116(3):481–485. doi:10.2109/jcersj2.116.481

[22] Monma H. The incorporation of dicarboxylates into octacalcium bis(hydrogen phosphate) tetrakis(phosphate) pentahydrate. Bull Chem Soc Jpn. 1984;57(2):599–600.

[23] Yokoi T, Nakamura J, Ohtsuki C. Incorporation behavior and biomedical applications of inorganic-layered compounds. In: Osaka A, Narayan R, editors. Bioceramics from macro to nanoscale. Amsterdam (Netherlands): Elsevier; 2020. p. 139–158.

[24] Yokoi T, Kawashita M. Understanding the steric structures of dicarboxylate ions incorporated in octacalcium phosphate crystals. Materials. 2021;14:2703. 34063897 10.3390/ma14112703 PMC8196614

[25] Fowler BO, Marković M, Brown WE. Octacalcium phosphate carboxylates. 3. Infrared and Raman vibrational spectra. Chem Mater. 1993;5:1417–1423.

[26] Marković M, Fowler BO, Brown WE. Octacalcium phosphate carboxylates. 2. Characterization and structural consideration. Chem Mater. 1993;5(10):1406–1416.

[27] Laurencin D, Li Y, Duer MJ, et al. A ^43^Ca nuclear magnetic resonance perspective on octacalcium phosphate and its hybrid derivatives. Magn Reson Chem. 2021;59:1048–1061. 33729624 10.1002/mrc.5149

[28] Li Y, Reid DG, Duer MJ, et al. Solid state NMR -an indispensable tool in organic-inorganic biocomposite characterization; refining the structure of octacalcium phosphate composites with linear metabolic di-acids succinate and adipate. Solid State Nucl Magn Reson. 2018;95:1–5. 30170130 10.1016/j.ssnmr.2018.08.004 PMC6181798

[29] Tsai TWT, Chou FC, Tseng YH, et al. Solid-State NMR study of octacalcium phosphate incorporated with succinate. Phys Chem Chem Phys. 2010;12:6692–6697. 20422114 10.1039/b923338e

[30] Yokoi T, Goto T, and Sekino T, et al. Fluorescent properties of octacalcium phosphate with incorporated isophthalate ions. J Ceram Soc Jpn. 2022;130(5):337–340. doi:10.2109/jcersj2.21173

[31] Tsunawaki Y, Sakamoto K, Yamaguchi S, et al. Contribution of microwave to the formation of octacalcium phosphate intercalating succinate ions. J Infrared Millim Terahertz Waves. 2021;42:409–415.

[32] Yokoi T, Goto T, Hara M, et al. Incorporation of tetracarboxylate ions into octacalcium phosphate for the development of next-generation biofriendly materials. Commun Chem. 2021;4(1):4. doi:10.1038/s42004-020-00443-5 PMC9814588 36697512

[33] Sugiura Y, Makita Y. Ammonium inhibition of the intercalation of dicarboxylic acid molecules into octacalcium phosphate layer by substitution. J Solid State Chem. 2019;279:120923.

[34] Yamada I, Tagaya M. Immobilization of 2,2’-bipyridine-5,5’-dicarboxylic acid in layered octacalcium phosphate. Colloid Interface Sci Commun. 2019;30:100182.

[35] Yokoi T, Machida S, Sugahara Y, et al. Enantioselective incorporation of dicarboxylate guests by octacalcium phosphate. Chem Commun. 2017;53(48):6524–6527. doi:10.1039/C7CC01169E 28573292

[36] Yokoi T, Kamitakahara M, Ohtsuki C. Continuous expansion of the interplanar spacing of octacalcium phosphate by incorporation of dicarboxylate ions with a side chain. Dalton Trans. 2015;44:7943–7950. 25826693 10.1039/c4dt03943b

[37] Davies E, Müller KH, Wong WC, et al. Citrate bridges between mineral platelets in bone. Proc Natl Acad Sci USA. 2014;111(14):E1354–E1363. doi:10.1073/pnas.1315080111 24706850 PMC3986129

[38] Yokoi T, Kamitakahara M, and Kawashita M, et al. Formation of organically modified octacalcium phosphate in solutions containing various amounts of benzenedicarboxylic acids. J Ceram Soc Jpn. 2013;121(2):219–225. doi:10.2109/jcersj2.121.219

[39] Yokoi T, Kato H, Kim IY, et al. Synthesis of octacalcium phosphate with incorporated succinate and suberate ions. Ceram Int. 2012;38(5):3815–3820. doi:10.1016/j.ceramint.2012.01.030 22249371

[40] Yokoi T, Kato H, Kim IY, et al. Formation of octacalcium phosphate with co-incorporated succinate and suberate ions. Dalton Trans. 2012;41:2732–2737. 22249371 10.1039/c2dt11580h

[41] Yokoi T, Kawashita M, Ohtsuki C. Effects of monocarboxylic acid addition on crystallization of calcium phosphate in a hydrogel matrix. IOP Conf Series Mater Sci Eng. 2011;18(19):192012.

[42] Yokoi T, Kato H, and Kamitakahara M, et al. Formation of octacalcium phosphate with incorporated succinic acid through gel-mediated processing. J Ceram Soc Jpn. 2010;118(6):491–497. doi:10.2109/jcersj2.118.491

[43] Sakamoto K, Yamaguchi S, Kaneno M, et al. Synthesis and thermal decomposition of layered calcium phosphates including carboxylate ions. Thin Solid Films. 2008;517(4):1354–1357. doi:10.1016/j.tsf.2008.09.050

[44] Nakahira A, Aoki S, Sakamoto K, et al. Synthesis and evaluation of various layered octacalcium phosphates by wet-chemical processing. J Mater Sci Mater Med. 2001;12:793–800. 15348226 10.1023/a:1017968818168

[45] Markovic M. Octacalcium phosphate carboxylates. In: Chaw L, Eanes E, editors. Octacalcium phosphate. Basel (Switzerland): Karger; 2001. p. 77–93. 10.1159/000061649 11758449

[46] Aoki S, Sakamoto K, Yamaguchi S, et al. Syntheses of octacalcium phosphate containing dicarboxylic acids and effects of the side groups on the crystal growth of octacalcium phosphate. J Ceram Soc Jpn. 2000;108:909–914. Japanese

[47] Kijima T, Yamaguchi K, Miyata A, et al. Crystallization of Calcium Phosphate Templated by α-Amino Acids Depending on Their Composition, Chain Length, and Enantiomerism. Chem Lett. 2000;29(11):1324–1325. doi:10.1246/cl.2000.1324

[48] Monma H. Apatitic intercalation compounds containing dicarboxylates. Gypsum Lime. 1992;237: 108–114. Japanese.

[49] Aoki S, Nakahira A, Nakayama H, et al. Synthesis and aldehyde absorption properties of aspartate-octacalcium phosphate inclusion compound. J Phys Chem Solids. 2004;65(2–3):465–470. doi:10.1016/j.jpcs.2003.10.030

[50] Tuncer M, Bakan F, Gocmez H, et al. Capacitive behaviour of nanocrystalline octacalcium phosphate (OCP) (Ca_8_(H_2_(PO_4_)_6_‧5H_2_O) as an electrode material for supercapacitors: biosupercaps. Nanoscale. 2019;11:18375–18381. 31573596 10.1039/c9nr07108c

[51] Kawai T, Echigo S, Matsui K, et al. First clinical application of octacalcium phosphate collagen composite in human bone defect. Tissue Eng Part A. 2014;20(7–8):1336–1341. doi:10.1089/ten.tea.2013.0508 24294829 PMC3993018

[52] Ishihara S, Matsumoto T, Onoki T, et al. New concept bioceramics composed of octacalcium phosphate (OCP) and dicarboxylic acid-intercalated OCP via hydrothermal hot-pressing. Mater Sci Eng C. 2009;29(6):1885–1888. doi:10.1016/j.msec.2009.02.023

[53] Yokoi T, Goto T, Kato T, et al. Hydroxyapatite formation from octacalcium phosphate and its related compounds: a discussion of the transformation mechanism. Bull Chem Soc Jpn. 2020;93:701–707.

[54] Yamada I, Noda D, Shinozaki K, et al. Synthesis of luminescent Eu(III)-doped octacalcium phosphate particles hybridized with succinate ions and their reactive behavior in simulated body fluid. Cryst Growth Des. 2021;21:2005–2018.

